# Strategies for Selecting Membrane Protein-Specific Antibodies using Phage Display with Cell-Based Panning

**DOI:** 10.3390/antib6030010

**Published:** 2017-08-05

**Authors:** Mohamed A. Alfaleh, Martina L. Jones, Christopher B. Howard, Stephen M. Mahler

**Affiliations:** 1 Australian Institute for Bioengineering and Nanotechnology (AIBN), The University of Queensland, Brisbane, Queensland 4072, Australia; c.howard2@uq.edu.au (C.B.H.); s.mahler@eng.uq.edu.au (S.M.M); 2 Faculty of Pharmacy, King Abdulaziz University, Jeddah 21589, Saudi Arabia; 3 Australian Research Council Training Centre for Biopharmaceutical Innovation, The University of Queensland, Brisbane, Queensland 4072, Australia; 4 Centre for Advanced Imaging, The University of Queensland, Brisbane, Queensland 4072, Australia

**Keywords:** affinity selection, antibodies, cell markers, cell receptors, competitive elution, epitopes, panning, phage display, transfection, whole cell

## Abstract

Membrane proteins are attractive targets for monoclonal antibody (mAb) discovery and development. Although several approved mAbs against membrane proteins have been isolated from phage antibody libraries, the process is challenging, as it requires the presentation of a correctly folded protein to screen the antibody library. Cell-based panning could represent the optimal method for antibody discovery against membrane proteins, since it allows for presentation in their natural conformation along with the appropriate post-translational modifications. Nevertheless, screening antibodies against a desired antigen, within a selected cell line, may be difficult due to the abundance of irrelevant organic molecules, which can potentially obscure the antigen of interest. This review will provide a comprehensive overview of the different cell-based phage panning strategies, with an emphasis placed on the optimisation of four critical panning conditions: cell surface antigen presentation, non-specific binding events, incubation time, and temperature and recovery of phage binders.

## 1. Monoclonal Antibodies

Monoclonal antibodies (mAbs) are biological macromolecules derived from B-lymphocytes. The specificity and high affinity of a mAb towards a particular target makes them attractive drug candidates within the biopharmaceutical industry. Their success as therapeutic entities is due to their proven efficacy in targeted therapy, ability to be structurally modified and relatively low toxicity profile in relation to other small molecule pharmaceuticals [1,2]. To date, more than 40 mAbs have been approved for clinical applications and many more are under clinical and pre-clinical investigation [3,4].

### 1.1. Generation of Monoclonal Antibodies Using Hybridoma Technology

Monoclonal antibodies were first produced using hybridoma technology in which an immortal myeloma cell is fused with an antibody-producing spleen cell of an immunized animal [5,6]. Although revolutionary and still commonly used to produce antibodies as research reagents, clinical applications of murine-derived mAbs have limited therapeutic efficacy due to the immune response that is mounted against these mAbs by the patient. Antibody engineering techniques were subsequently utilised to create chimeric or humanized antibodies by utilising the murine variable regions or complementary determining regions, respectively, in conjunction with human constant regions, in order to reduce the human anti-mouse antibody response (HAMA) [7,8,9]. Fully human antibodies are now generated using the same hybridoma technology in transgenic mice that have human immunoglobulin loci [10]. Antibody phage display is an alternative technique for generation of monoclonal antibodies, whereby antibodies can be isolated from large immunoglobulin gene repertoires [11] rapidly in a matter of weeks.

### 1.2. Generation of Monoclonal Antibodies Using Phage Display Technology

Phage display, first described by George P. Smith in 1985, is a powerful tool for displaying proteins or peptides of interest on filamentous phage through fusion with a viral coat protein [12]. Each phage clone within an antibody phage library displays an exclusive antigen-binding site, made up of random combinations of heavy and light chain variable fragments cloned from pools of B-cells [13]. Several binding entities such as variable domain (Fv; variable regions of the heavy (VH) or variable regions of the light chain (VL)), single-chain variable domain (scFv), fragment antigen binding (Fab) or peptides that might serve as novel therapeutics have been isolated from phage display libraries [14,15,16,17,18]. Antibody phage libraries are constructed from mRNA extract from natural sources, such as bone marrow, peripheral lymphocytes or the spleen [19,20], which can be either immunized [21,22] or naïve [23]. Alternatively, phage libraries could be synthetic in order to enhance the antibodies’ characteristics or to make a large and highly diverse phage repertoire. Creating combinatorial antibody libraries on filamentous phage have been achieved through combined populations of VH- and VL-domains which are joined by a flexible protease-resistant glycine-serine linker (Gly4Ser)_3_ into a single DNA sequence [14]. Monovalent display is the most popular display system because it allows for selection of higher affinity antibodies [24,25,26,27].

Antibody display also includes other newer technologies such as yeast and mammalian display. Wittrup and Boder were the first to describe the yeast surface display system when they fused scFv antibody fragments to the C-terminus of the binding subunit of *Saccharomyces cerevisiae* a-agglutinin receptor (Aga2p) [28]. Since then, different yeast antibody libraries were constructed to display full-length antibodies as well as antibody fragments such as scFvs and fragment antigen-binding region (Fabs) [28,29,30]. Several antibodies have also been successfully isolated against different targets through yeast display [31,32,33,34]. Additionally, mammalian cells have been used to display scFv or whole Immunoglobulin Gs (IgGs) [35,36,37,38,39,40], allowing isolation of high affinity antibodies with specific biological functions [37,38]. Each display system has its advantages and disadvantages, however, determining those are beyond the scope of this review. Here, we will instead focus on the most popular type of antibody display, the phage display.

In phage display, filamentous phage strains such as M13, f1 and fd are the most commonly used types of phage, since they are stable, easy to manipulate and capable of keeping the infected cells intact during their life cycle [41,42,43,44]. Filamentous phage is a flexible cylindrical shaped virus particle that consists of circular single-stranded DNA (6407-base), coated with five different coat proteins (pIII, pVIII, pVI, pVII and pIX) [45,46]. Although all the aforementioned coat proteins can be fused with a product to construct phage display libraries, pIII-fused products are the most commonly used. The pIII is able to display large protein sequences such as scFv and Fab [44,47,48], and the crystal structure of pIII shows that it is willing to accept peptide or protein without losing its function, due to the structural flexibility [49].

There are more than 100,000 publications and 80,000 patents related to phage display documented in the three decades since Smith’s seminal report in 1985 [50]. Some blockbuster drugs, with annual sales beyond US$1 billion, such as Lucentis and Humira, have been isolated using phage display [51]. The latter is considered the highest selling drug globally, with total sales of US$11 billion since it was launched in 2002 [51]. Now, approximately 35% of mAbs in clinical trials are derived from phage display, which means phage display will remain a prominent platform technology for mAb discovery in the biologics industry [50,51].

Panning (also called biopanning) or affinity enrichment is a technique for the isolation of antibody fragments from a phage library based on their binding affinity to a given target [52]. The general procedure includes three major steps used for selection: (i) exposing the library to the desired target and capturing specific phage binders; (ii) washing to eliminate non-selective or low affinity binders; and (iii) eluting the specific binders and amplification of the recovered phage. This process is usually repeated in an iterative manner three to six times, in which the diversity of phage is reduced while the proportion of selective strong affinity binders to the target is increased [53,54,55]. Although it is a fairly straightforward technique, there are many factors that can influence the overall outcome, such as library quality, nature of the target, binding affinity, change of pH and other environmental conditions, and availability of the desired binder within the library. One of the most important factors to consider is how to present the antigen to the library to ensure it is displayed in its correct conformation. Direct immobilisation of purified antigen onto plastic is the most common method, but this may result in conformational changes of the target antigen. Other methods include solution-based panning using biotinylated antigens [56] or calmodulin binding peptide-tagged antigen [57] or panning on the surface of whole cells. Cell-based panning allows the antigens to sustain their natural conformation, this ensuring the isolated binders are able to access epitopes in vivo. Cell-based panning may be used to identify new cell biomarkers by isolating antibodies which can discriminate between different cellular states, or it can allow antibody isolation even when the antigen of interest in not available in pure form [58].

## 2. Whole Cell-Based Phage Display: A Monoclonal Antibody Discovery Platform for Membrane Proteins

Membrane proteins (e.g., G protein-coupled receptors, ligand-gated ion channels, receptor tyrosine kinases and immunoglobulin-like receptors) are desirable targets for phage display in order to obtain novel mAbs for research, diagnostic and therapeutic purposes. They are a large group of proteins that play key roles in transportation and signal transduction, and account for 20 to 30% of all proteins in most living entities [59,60]. In diseases such as cancer, some membrane receptors change their normal activity levels, for instance, overexpression of human epidermal growth factor receptor 2 (Her2) in breast cancer [61] and Mesothelin in malignant pleural mesothelioma [62]. Membrane receptors represent 44% of human drug targets, which make them the largest group of drug targets [63,64].

Despite huge advancements in protein engineering and manufacturing, expression of membrane proteins in a soluble form and subsequent purification are still challenging and time-consuming. For some membrane proteins, it is simple to purify just the extracellular domain and successfully obtain antibodies using panning on immobilised protein [65,66,67,68]. However, many membrane proteins, such as ion channels, do not have a large extracellular domain that can be purified. Expressing these membrane proteins in large amounts is difficult and, outside of the hydrophobic environment of the cell membrane, these proteins can undergo conformational changes and/or aggregation. For instance, the expressed full-length soluble membrane proteins, such as the large family of the 7-transmembrane (7-TM) G-protein-coupled receptors, which represent 12% of drugs targeting receptors [64], lack appropriate post-translational modifications and are poorly soluble in an aqueous media, because they contain complex structures that are not properly formed during recombinant expression [69]. They can easily form aggregates, lose their natural conformational structures and tend to denature when coated on solid surfaces. This means they become structurally different from what exists in nature [70,71,72], which consequently might lead to isolating binders that do not identify the antigen in its native form. Such binders might identify epitopes that could be masked or not exposed naturally and therefore not accessible on the antigen’s native conformational structure [73,74]. Different membrane protein presentation methods to overcome these issues have been reviewed by Huang et al. [75]; however, here we will focus on the various attempts to optimise whole cells displaying the antigen of interest, using native cells or engineered cell lines, to ensure the correct conformational structure, in a process called cell-based panning.

Screening antibody libraries against membrane proteins in their native or even near-native conformations using whole-cells or tissue to isolate mAbs might result in more biologically relevant antibodies which depend on binding to a specific antigenic determinant that is naturally exposed on the targeted protein [76,77,78]. Cell-based panning has many advantages over purified protein panning, since it can be employed to retrieve antibody clones that are specific for either known or unknown antigens that are present on the surface of specific cell populations; also it can be employed in case of unavailability of the targeted antigen in pure form [58,79,80,81].

Panning on a transfected cell line permits selection of antibodies to cell bound proteins without the need for purification of the desired protein, which as a result accelerates the generation of antibodies dramatically. This was evident in attempts to obtain binders to innate immunity receptor Toll-like receptor 2 (TLR2), a large complex membrane protein with a large extracellular domain [74]. The researchers used both standard soluble antigen-based and cell-based panning protocols on transfected cells in parallel for comparison purposes. None of the isolated clones obtained from the purified, recombinant protein strategy were able to bind the receptor in its native state on the cell surface. On the other hand, eight unique phage-derived binders from panning an immune library on transfected cells were able to bind TLR2 in its native structure.

Phage display panning can be also performed on yeast cells expressing the membrane bound protein on their cell surfaces. For instance, a high throughput approach was reported for generating several mAbs from a scFv antibody library against multiple different types of membrane proteins displayed on yeast surface [82]. Additionally, panning on yeast cells can be performed in combination with panning on mammalian cells, having both cell types expressing the same antigen of interest to enhance the selection process by directing the antibody-phage binders toward the targeted antigen [83]. Several specific antibodies were isolated after panning a scFv phage library against a breast cancer cell line overexpressing cell surface adhesion receptor (CD44) and ephrin type-A receptor 2 (EphA2) antigens for two rounds before panning the retrieved polyclonal phage pool on yeast expressing domain 1 of CD44 and the extracellular domain of EphA2 for another two rounds.

Despite the advantage of not requiring purified antigens, there are many difficulties associated with whole cell panning including low receptor density, high background of irrelevant antigens and non-specific adsorption of phage particles. Therefore, optimisation of the cell-surface antigen presentation, washing procedures, phage recovery and incubation conditions can contribute to a successful cell-based panning strategy.

## 3. Optimisation of Strategies for Phage Display with Cell-Based Panning

### 3.1. Optimising Cell Surface Antigen Presentation

The first and the most critical step in any cell-based panning method is the appropriate exposure of target antigen on the cell surface. Native cells can be used as an antigen source; for example, cancer cells can be incubated with a library that has first been depleted against the equivalent non-cancerous cells to identify novel cancer-specific reagents. However, native cells usually have relatively low expression levels of desired antigens which make it difficult to capture even high-affinity clones from a highly diverse library. In addition, assessing the optimal culturing conditions for native cells prior to panning is crucial to achieve higher antigen density on the cell surface. For instance, some native cell lines showed a drastic enrichment of certain cells markers when they grew under non-adherent sphere-forming culture conditions compared to traditional monolayer culture [84]. For known antigens, transfection and expression of recombinant protein on the cell surface allows for a higher antigen density. Examples of transfected cell pannings include stable transfection, (e.g., tetraspanin cell surface glycoprotein CD9 [85], müllerian inhibiting substance type II receptor (MISIIR) [86] and TLR2 [74]) and transient transfection, (e.g., canine tyrosine-protein kinase receptor CD117 (c-Kit), human CD83 and bat CD11b) [87].

Many studies show the ability to isolate unique binders after panning phage libraries against either confluent attached monolayers [88,89,90] or against cells in suspension [85,91]. However, in the case of CD146 and CD36 targets, successful isolation of binders was only achieved when the phage libraries were biopanned against cell lines in suspension rather than on monolayers [92,93]. Reportedly, the binders isolated from panning on confluent monolayers were specific to the culture flask plastic surface and the serum protein within the culture medium. Suspension culture has the additional advantage of a greater available surface area for target membrane protein presentation.

**Figure 1:**
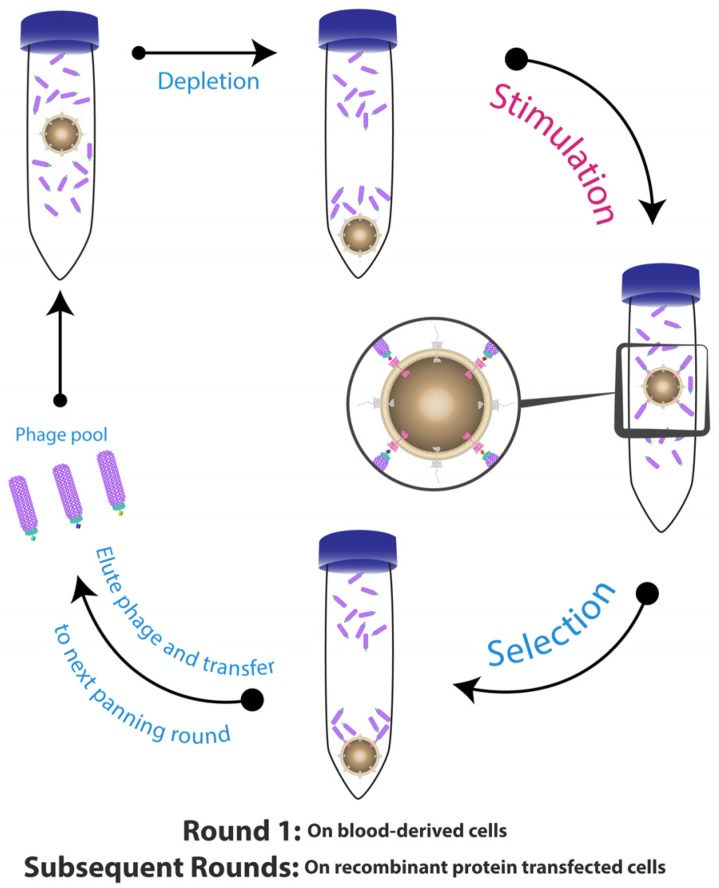
Selection of conformation-specific single-chain variable domain (scFv)-phage. The process of the panning started with depleting the scFv-phage library on non-activated cells having unwanted and non-stimulated epitopes. The non-specific scFv-phage/non-activated cell complex were centrifuged and discarded, while the supernatant containing the free scFv-phage were transferred into physiologically stimulated cells expressing neo-epitopes in their active conformational structure. The scFv-phage/activated cell complexes were centrifuged and the bound scFv-phage were both eluted with low pH or by competition with ligand, and collected for the subsequent panning rounds. Figure shows methodology described by Eisenhardt et al. [94].

Eisenhardt et al. described a cell suspension-based panning strategy for the selection of scFv-binders to a specific conformational state of a cell surface receptor (Figure 1) [94]. This protocol allows for isolation of scFv-antibodies against conformation-specific neo-epitopes that are exposed only after physiological alterations, which activated the receptors (platelet integrin αIIbβ3 (also known as Glycoprotein IIb/IIIa) and Macrophage-1 (Mac-1)) [95,96,97]. Briefly, this protocol involves firstly depleting the library on non-activated cells, then transferring the unbound phage to an activated cell suspension expressing the targeted receptors in their activated conformation. They also used a native cell line in the first round, and then changed to recombinant cells expressing the same receptor to reduce the non-specific binding background and improve the selection process.

In the panning strategies described above, relatively large numbers of cells (between 10^5^ and 10^7^), are required to provide sufficient display of the target antigen to the library. However, obtaining such large cell numbers can be impractical for native tissue, where only limited material from the patients may be available. A microfluidic technology or a microselection strategy can be applied to bypass the need for high cell numbers during the panning process.

A microfluidic phage selection approach offers a controlled, continuous fluid flow of phage-displayed peptides over live, adherent cells within a microfluidic chamber. This allows for both a high ratio of phage particles to cells, and a means for high stringency washing without cell loss [98]. Dorfmueller et al. [99] used the aforementioned microfluidic device to isolate phage-displayed antibodies against unknown cell surface markers that express in human corneal endothelial cells (hCECs). They successfully isolated a scFv specific to the hCECs layer and by integrating immunoprecipitation and mass spectrometry, they were able to identify immunoglobulin-like protein ALCAM (CD166) as a marker for hCECs. Microfluidics also offers a unique platform for automating the selection process, achieving high-throughput selection [100] and providing a robust continuous washing system that markedly reduces the rebinding events [101,102]. However, microfluidic channels are known for their ability to induce shear stress, which in some cell types can alter their physiological nature due to their sensitivity, such as endothelial cells [103,104].

**Figure 2:**
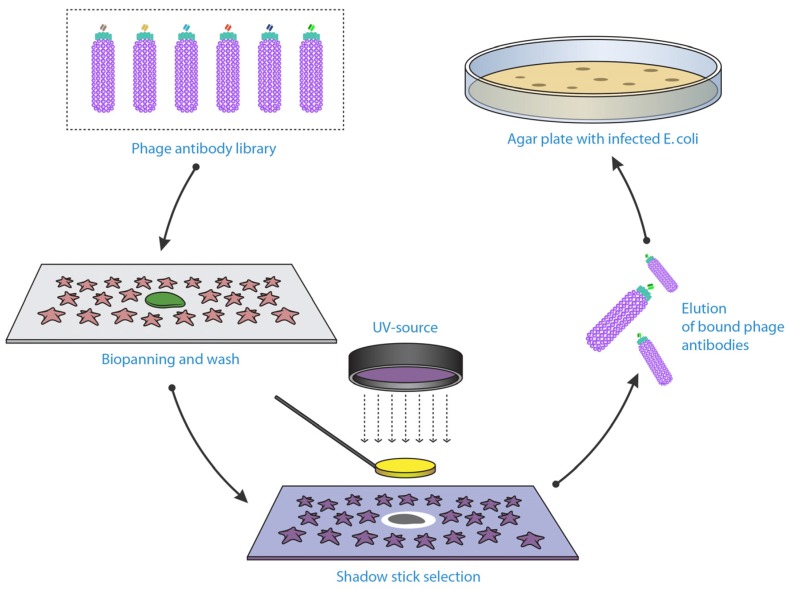
Isolating phage antibody binders against a single rare cell using shadow-stick selection technique. Cells placed on glass slide, with the targeted cell marked. Shadow-stick (minute flat gold disc attached to a glass pipette) placed on top of the targeted cell to protect the phage bound to it, before killing the unprotected phage with ultraviolet (UV) irradiation. Figure shows methodology adapted from Sanchez-Martin et al. [108].

Microselection or shadow-stick selection is an exceptional method that is able to retrieve unique binders by applying the phage library against rare cells within a heterogeneous solution (Figure 2) [105,106]. Sørensen et al. conducted the microselection by fixing the cell suspension sample onto a specific glass slide, with the rare cell marked according to its distinct morphology or by immunostaining if specific biomarkers are known. This is followed by application of a scFv-phage library and subsequent washing steps. The unique cell was relocated and shielded with a minute flat gold disc attached to a glass pipette “shadow stick” before inactivating all the unshielded phage with ultraviolet (UV) irradiation. The only viable phage remaining were those bound to the target cell; these were then eluted and amplified. This method can help to identify novel biomarkers that are present on these rare cells. Microselection to isolate antibodies specific for very rare cell types, has potential for enhancing current diagnostic procedures, since currently unique cells types such as circulating tumour cells, are very difficult to detect due to their very low percentage in patients’ peripheral blood [107].

Even in a seemingly optimised cell-based panning method, the nature of the antigen highly influences the likelihood of success. Hoogenboom et al. performed identical cell based panning procedures on two different human receptors, somatostatin and CD36 expressed on Chinese hamster ovary (CHO) cells, in an attempt to isolate antibodies that are able to bind epitopes within their extracellular domains [93]. They were unable to isolate any somatostatin receptor-specific phage clones even when using ligand-competitive elution. Somatostatins have short, heavily glycosylated extracellular loops of ~40 amino acids and have low immunogenicity, which make them challenging targets [93,109]. On the other hand, they were successful in isolating antibodies against CD36, which is characterised by its large, highly glycosylated immunogenic extracellular domain, which proved to be a straightforward target for cell based panning [93,110]. Jones et al. [87] also alluded to the success of cell-based phage panning being very antigen-dependent, having achieved greater success with large elongated receptor CD117 compared with shorter compact CD11b. These results confirm that the nature and the accessibility of the targeted membrane-bound receptors can determine the cell-based panning outcomes.

### 3.2. Reducing Non-Specific Binding Events

The approach of panning on whole cells or tissue is a notoriously tedious process. Antigens in their natural conformation are complex and exist at lower concentrations on the cell surface as compared to the rest of the host cell proteins, and the phage are therefore exposed to a population of antigens which are mostly irrelevant, both on the inside and the outside of the cell. The ultimate goal therefore is to enhance the selection efficiency of mAbs identifying only the desired surface antigens against a background of irrelevant antigens.

**Figure 3:**
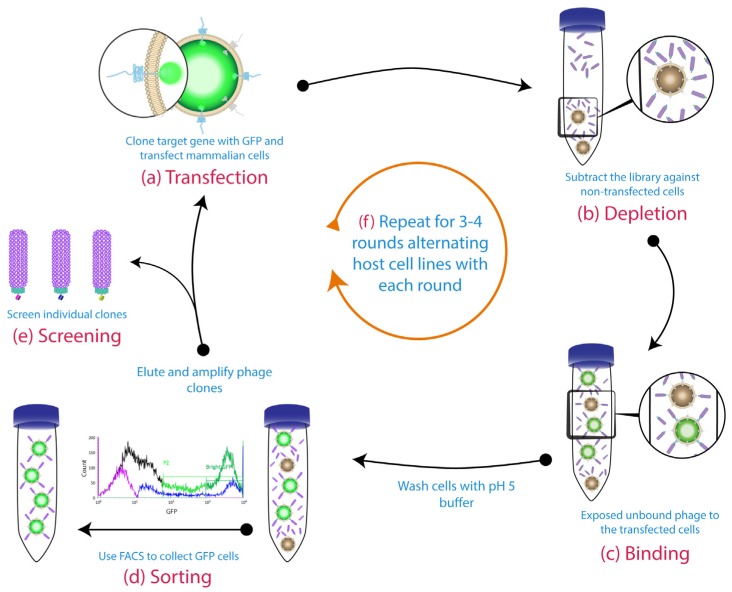
Cell-based panning protocol using GFP-sorting and alternative host cell lines: (**a**) The gene for the target membrane protein was cloned in-frame with GFP to be expressed on the cell surface. (**b**) The phage library was subtracted on untransfected cells. (**c**) Then, the free phage were panned against transiently transfected cells. A pH 5 wash step was conducted before the fluorescence-activated cell sorting method (FACS) sorting (**d**). The binders from the sorted cells were eluted with a low pH buffer. These binders were then amplified for the subsequent rounds and individual clones were screened by the end of biopanning campaigns (**e**). (**f**) The host cell line was alternated each round (1st round: Chinese hamster ovary (CHO) cells, 2nd round: human embryonic kidney 293 cells (HEK293), etc.). Figure shows methodology described by Jones et al. [87].

The use of blocking agents, such as Bovine Serum Albumin (BSA), milk and casein is a common practise in phage display to block the non-specific sites and thereby prevent the non-specific binding events [111,112]. However, in cell-based phage display, it is also critical to deplete the library of binders to other non-relevant cell-surface antigens by exposing the library on a similar cell line which is negative for the desired antigen, prior to the positive cell line [88,91,113,114,115]. If possible, alternating cell lines expressing the same target antigen can help to eliminate any irrelevant binders that are enriched in the first round (Figure 3) [87]. This strategy is possible when using transfected cells, as the host cell can easily be changed each round. Alternatively, switching the panning each round between latex beads coated with the purified form the antigen and cells expressing the same antigen, would enhance the selection procedure [116].

A cell suspension used for panning contains not only the fully intact healthy cells that express the antigen of interest, but it also contains dead cells, cell debris and cells that do not express the targeted protein on their surfaces, which can often lead to isolation of non-specific binders. De Kruif et al. was the first to report a subtractive process to rapidly isolate phage-derived binders against B-cell specific markers in their native conformation, by incorporating a fluorescence-activated cell sorting method (FACS) using anti-CD3 and anti-CD20 fluorochrome-labelled antibodies to separate subpopulations of T lymphocytes (CD3+) and B lymphocytes (CD20+) from peripheral blood cells [117].

However, this sorting method is unable to distinguish between cells harbouring the desired tumour-associated antigens from others within the same cell subpopulation, which might result in isolation of undesirable binder candidates. This issue has been overcome by incorporating detectable tags into the target antigen. For example, cells expressing human Müllerian inhibiting substance type II receptor (MIS-IIR) tagged with Hemagglutinin (HA) (detectable with fluorescein-conjugated anti-HA antibody) or canine CD117 fused with green fluorescent protein (GFP) can be isolated by FACS from a cell mixture after incubation with a phage library (Figure 3) [86,87]. Accordingly, several anti-MISIIR and anti-CD117 scFv clones were successfully isolated from the sorted antigen-expressing cells. According to Poul, typical cell-based panning strategies result in 1–12 phage bound per cell [115]. Using this modified–FACS technique resulted in two phage bound per cell after three rounds, which mean specific binders with less background of non-specific binders [86].

Another study described an effective method that dramatically improves the selection of antibodies against cell surface antigens rather than intracellular antigens by eliminating the dead cells and cell debris from the cell suspension before each panning round [118]. It was achieved by incubating breast carcinoma cell (MCF7) with biotinylated Annexin V, which binds to the intracellular phospholipid membrane phosphatidylserine when it is translocated to the cell surface in dead or dying cells [119]. The biotinylated Annexin V bound cells were then captured by streptavidin linked to Dynabeads (superparamagnetic spherical polymer particles) [118,120,121] to remove dead cells and cell debris. As a result, the phage library was exposed only to live cells, ensuring that the isolated phage will bind to antigens on the cell surface.

The non-specific background not only includes the undesirable cellular compartments and irrelevant cell-surface antigens, but also the phage that are non-specifically adhered to the cell surfaces via interaction with the phage coat proteins. These phage will be propagated along with the specific phage making enrichment of the correct clones difficult. Amongst the non-specifically bound phage will be a considerable population of phage which are not displaying an antibody fragment, due to insert-free phagemid vectors being present in the library [122,123]. Insert-free vectors arise from the incomplete restriction and/or re-ligation of the phagemid during antibody phage library cloning. Insert-free phage will have a growth advantage during phage amplification as they are not expressing the scFv-g3p fusion protein, and therefore they become enriched over several rounds of panning [122,123,124]. Two methods have been established to address the problem of non-specific adherence of phage to cell surfaces. Firstly, the interaction of phage with cell surfaces via coat proteins can be reduced by 75% by conjugating polyethylene glycol (PEG) polymers to the phage particles [125]. This creates a hydration sphere reducing non-specific interaction. Secondly, non-specifically adhered phage can be removed by incorporating a low pH buffer (pH 5.0) into the washing steps [124]. Removal of non-specific phage was demonstrated by showing that the enrichment of insert-free phage was prevented when this wash was incorporated [124].

**Figure 4:**
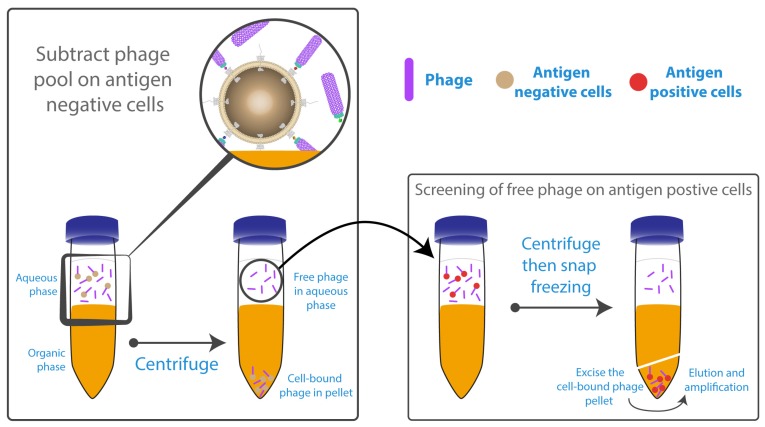
Whole cell-based panning using Biopanning and Rapid Analysis of Selective Interactive Ligands (BRASIL). Panning started with a depletion step for the phage library on cells not expressing the antigen of interest. The phage/unwanted cells complex were centrifuged into the organic phase. Free phage within the aqueous supernatant are transferred and incubated with cells expressing the antigen of interest into the aqueous solution before centrifugation through the organic phase. Bound phage were rescued from the organic phase, and then amplified to be used for the subsequent panning rounds. Figure shows methodology described by Giordano et al. [126].

One of the most critical drawbacks of panning methodology is the repeated washing and centrifugation steps required to remove non-specifically bound phage. In some cases, it can be up to 10 times per round [90], which might lead to loss or lysis of cells, especially in the presence of surfactant such as Tween-20 in the washing buffer. Optimised washing conditions have not been fully investigated and there are few studies addressing this particular issue. A technique known as BRASIL (Biopanning and Rapid Analysis of Selective Interactive Ligands) has been developed by Giordano et al. [126] and utilised successfully by Lipes et al. [74] and Carneiro et al. [127], eliminating the need for multiple washing steps (Figure 4). BRASIL is a simple, sensitive, single-centrifugation step that separates phage-cell complexes from unbound phage by differential centrifugation. This is achieved by adding a non-miscible organic solvent, to the aqueous cell-phage suspension. After centrifugation, two separate phases are formed; the cells with bound phage form a pellet in the non-miscible lower hydrophobic phase, while the upper hydrophilic phase contains the unbound phage. Centrifuging the cells through the non-polar solution has the additional advantage of effectively removing any loosely bound phage. Nevertheless, it cannot guarantee the complete removal of undesirable specific phage/antigen binding because the cell pellet within the organic phase also contains unwanted cellular compartments.

### 3.3. Optimising Incubation Time and Temperature

Temperature and incubation time can affect the kinetic properties of antibodies during the selection process. For instance, in an experiment to obtain antibodies against *Chlamydophila psittaci* (*C*. *psittaci*), no binders were isolated when the panning was performed at 4 °C, but enrichment of binders was observed when the panning was performed at 37 °C [128]. However, most of the published protocols recommend performing the selection step at 4 °C for 2–16 h (less incubation time is recommended) for membrane receptors, because at low temperature the receptor-mediated internalization is remarkably reduced and the cells become desensitized to environmental stimuli [74,101,103,126,129]. However, unique phage-derived clones against membrane proteins have also been retrieved after incubating phage libraries with cells at 20 °C or 37 °C for 1–20 h [90,91,93]. In some cases, incubation at 4 °C might cause receptor clustering or unspecific cell activation, and might also hinder the expression of specific epitopes [94]. If it is necessary to perform the incubation step at room temperature, a short incubation period (2 h) is recommended to reduce the internalization rate [94].

**Figure 5:**
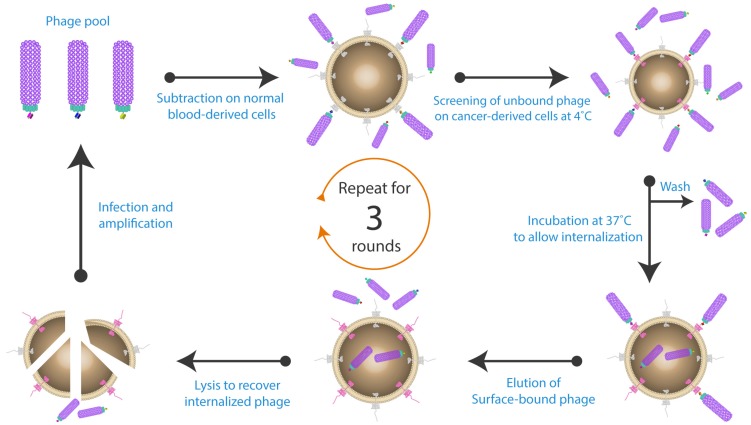
Isolation of specific internalizing antibodies by phage display. The antibody-phage library was initially subtracted on healthy cells, and then the free phage were screened on cells harbouring the antigen of interest. A washing step was performed to remove all the non-specific and unbound phage. All the previous steps were conducted at 4 °C. The cells were then incubated at 37 °C to induce phage internalization. Cell surface bound phage were removed by low pH elution buffer, while the internalized phage were retrieved by cell lysis. The isolated phage particles were then amplified and used for subsequent panning rounds. Figure shows methodology described by Fitting et al. [130].

In other cases, it is desirable to isolate antibodies that can internalize, to allow receptor-mediated endocytosis for targeted drug delivery. Poul et al. [115] and Fitting et al. [130], conducted panning using both 4 °C and 37 °C to isolate tumour specific antibodies that can be internalized (Figure 5). All the steps dedicated to remove the non-specific and weakly bound phage were performed at 4 °C to prevent receptor mediated internalization, followed by an incubation step at 37 °C for 15 min to stimulate surface-bound phage endocytosis. Reducing the incubation temperature to 4 °C after the short 37 °C incubation is crucial to avoid the degradation of endocytosed phage within the lysosome [131]. After removing the remaining surface bound phage by low pH elution, the internalized phage can then be retrieved from the cell lysate.

pH-sensitive dyes are only fluorescent when they internalize into biologically relevant low pH environments such as the intracellular compartment of the cell or in the intisticial space of the tumour [132,133,134]. Weissleder et al., were able to bioconjugate wild type filamentous bacteriophage with a water-soluble pH-sensitive dye that is fluorescent in acidic environments and non-fluorescent at neutral or basic pH [133]. Therefore, this approach can be employed on filamentous phage displaying antibody variable domains to obtain fluorescence-based images after panning on whole cells to identify the internalised binders. This technique can be utilised to adjust the required time of internalization during the incubation step at 37 °C and consequently determine the optimum duration of incubation.

### 3.4. Improving Phage Recovery

Usually, cell-bound phage are eluted by using a low pH buffer [90,91]. However, some studies indicate that acidic pH is not sufficient and a significant portion of phage still remain intact with the cell pellet after centrifugation [92,93]. Alternately, using an alkaline solution or direct infection of cell-bound phage with logarithmically growing *E. coli* might be reasonable alternatives for better phage recovery [74,92,93,126]. Other studies indicate that a significant portion of phage is internalized into the cells post-binding to cell surface antigens, so combining the low pH solution with or without non-denaturing detergents (e.g., NP-40 or Tween 20) will enhance the phage recovery, drastically [135,136]. These type of detergents can lyse the cells without affecting the natural structural configurations of the proteins [137].

Some protocols use a competitive elution strategy by using known physiological ligands or specific blockers to displace the bound phage. For example, Eisenhardt et al. used a range of competitive binders such as eptifibatide and abciximab for GPIIb/IIIa, or even known biological ligands such as fibrinogen for GPIIb/IIIa and Mac-1 were used [94]. While Hageman et al. used CCL17 and CCL22 (G-protein coupled chemokine receptor (CCR4)-specific chemokines) to isolate anti-CCR4 mAbs that were able to antagonize the receptor specific ligands [138]. Indeed, competitive elution strategy is valuable when panning on native cell lines, since they are naturally expressing multiple antigens on their surfaces. For example, the human bladder carcinoma cell line T24 expresses different cytokeratins (CKs) such as CK 7, 8 and 18 [139]. A competitive elution using anti-CK8 mAbs (RCK 102) was useful to elute phage binders specific to CK 8 on T4 cell line [140,141]. This method of elution allowed isolation of a large diversity of high affinity phage clones against CK 8, confirmed by their reactivity against CK 8 antigen in ELISA, immunoblotting and immunofluorescence assays [140,141]. Although this method seems feasible and effective, optimising the concentrations and incubation periods of competitive elution agents is critical to achieve the expected outcomes [141].

## 4. Conclusions

Several cell-based panning strategies have been published over the years and, here, we highlighted the differences and the novelty of many of them. There is no cell-based panning protocol which provides a fully optimised “plug and play” scheme that could be translated flawlessly to challenging or different membrane protein systems. The reviewed protocols offer valuable descriptions of the potential pitfalls that pertain to whole cell panning and provide suggestions to overcome several of them. It is clear that whole cell panning is challenging even for membrane proteins with relatively simple structures and large extracellular domains. More complex structures such as multi-pass ion-channels and G-protein coupled receptors present an even greater challenge as they present relatively little exposed epitope to the phage library. Technique optimisations must be done on a case-by-case basis and should focus on ensuring a high display level of correctly-folded target antigen, and removing non-specific binding events.

In brief, phage display is an inexpensive, facile and powerful tool in drug discovery and development that allows isolation of recombinant mAbs with high specificity against particular antigens in short period of time. There are several FDA approved phage display-derived antibodies and antibody fragments, such as adalimumab, ranibizumab and necitumumab, and many others in clinical trials, such as mavrilimumab and carlumab [142,143,144]. Many academic institutions and industrial laboratories are continually developing methods for the design, construction and screening of antibody libraries. Further improvements are expected to be achieved in the coming years as this technology contributes significantly toward therapeutic drug discovery. This review article presents several examples of cell-based panning strategies that have been successful for their particular application. However, there has been limited research to optimise the method of cell-based panning. Optimisations aim to improve the presentation of the antigen of interest, to reduce non-specific binding events, to increase the enrichment of desirable binders over those binding irrelevant antigens and to minimize cell loss. Methodology for cell-based panning is still being refined, with many novel techniques being described. A successful cell-based panning campaign is likely to be highly dependent on the antigen of interest, and the methodology needs to be optimised on a case-by-case basis.
